# Computational prediction and experimental validation of a novel synthesized pan-PIM inhibitor PI003 and its apoptosis-inducing mechanisms in cervical cancer

**DOI:** 10.18632/oncotarget.3139

**Published:** 2015-02-04

**Authors:** Zhongyu Liu, Weihua He, Jianglin Gao, Junhua Luo, Xian Huang, Chunfang Gao

**Affiliations:** ^1^ Anal-Colorectal Surgery Institute, No.150 Central Hospital of PLA, Luoyang, Henan 471031, China; ^2^ Department of Obstetrics & Gynecology, No.150 Central Hospital of PLA, Luoyang, Henan 471031, China

**Keywords:** PIM kinase family, Pan-PIM inhibitor (PI003), HeLa cell, Apoptosis, MicroRNA

## Abstract

PIM protein family, short-lived serine/threonine kinases (PIM1, PIM2 and PIM3), are weak oncogenes but contribute to tumorigenesis as cancer targets. Thus, design of a novel pan-PIM inhibitor is still a challenge for current cancer drug discovery. Herein, we used a Naïve Bayesian model to construct the PIM network and identified Bad and Hsp90 to interact with PIMs. Then, we screened a series of candidate small-molecule compounds targeting PIMs, and subsequently synthesized a novel small-molecule compound PI003 with remarkable anti-proliferative activities in cervical cancer cells. Moreover, we found that PI003 induced apoptosis via the death-receptor and mitochondrial pathways by targeting PIMs and affecting Bad and Hsp90. Combined with microRNA microarray analyses, we demonstrated that some microRNAs such as miR-1296 and miR-1299 could affect PIM1-STAT3 pathway in PI003-induced apoptosis. Finally, we reported that PI003 had remarkable anti-tumor activity and apoptosis-inducing effect in *in vivo* mouse model. In conclusion, these results demonstrate that PI003, as a novel synthesized pan-PIM inhibitor, induces the death-receptor and mitochondrial apoptosis involved in microRNA regulation, and also possessed remarkable anti-tumor activity and apoptosis-inducing effect *in vivo.* Thus, these findings would shed light on discovering more potential new small-molecule pan-PIM inhibitors in future cervical cancer therapy.

## INTRODUCTION

PIM proteins are a family of short-lived serine/threonine kinases that are highly evolutionarily conserved in multicellular organisms [1]. PIM kinase family is composed of three members, PIM1, PIM2, and PIM3, which are highly homologous at the amino acid level (PIM1 and PIM3 are 71% identical, whereas PIM1 and PIM2 share 61% homology), but differ partially in their tissue distributions [2]. Unlike other kinase activities, the activity of PIM kinases is not primarily regulated by phosphorylation; instead, PIM kinases are mainly regulated by transcription. And, PIM kinases do not have a regulatory domain and are constitutively active when expressed [3]. Thus, PIM kinases can appear to be regulated at the level of transcription, translation, and even proteasomal degradation [4].

PIM family members are weak oncogenes but can contribute to tumorigenesis by selectively enhancing tumorigenic capabilities [5]. This effect appears to depend on the tissue and the nature of the pathways activated by the molecularly cooperating oncogenes [6]. PIM kinases represent interesting drug targets because they are often overexpressed in many cancers and are involved in cancer-specific pathways, such as cell survival, cell cycle progression and cell migration. For instance, blocking PIM1 function via the introduction of a dominant-negative PIM1 sensitizes pancreatic cancer cells to apoptosis induced by glucose deprivation. Moreover, dominant-negative PIM1 reduces tumorigenicity in pancreatic cancer cells and HeLa xenograft mouse models [7, 8]. The emerging importance of PIM kinases in human tumorigenesis has increased interest in developing small molecule inhibitors targeting these proteins. Different classes of PIM inhibitors have recently been reported [9–11], but only a few of them have been tested in cell-based assays or animal models to demonstrate their anti-cancer activities. In addition, only a few of these inhibitors are effective against all PIM family kinases because most of them have been focused on PIM1 [12, 13]. Due to functional redundancy, simultaneous targeting of all PIM kinases can be advantageous in treating cancer patients. Thus, it will led to the design and synthesis of more new pan-PIM inhibitors for current and future cancer therapy.

In this study, we reported herein that PI003, as a novel synthesized pan-PIM inhibitor, could induce the death-receptor and mitochondrial apoptosis involved in some microRNA regulation, and also possessed remarkable anti-tumor activity and apoptosis-inducing effect *in vivo*.

## RESULTS

### *In silico* analysis of the PIM kinase family network

**Figure 1:**
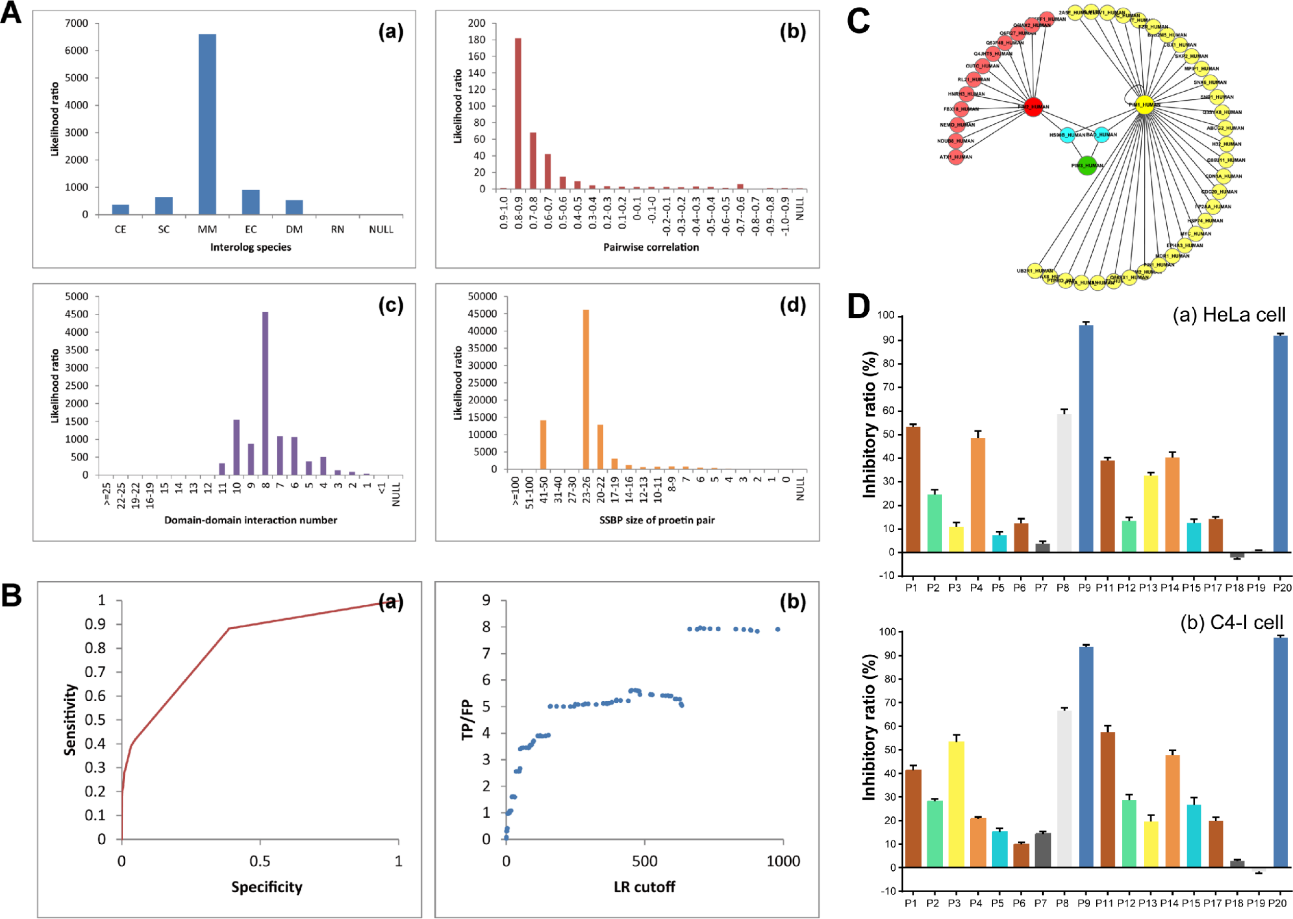
Network-based identification of PIM-modulated apoptotic pathways and screening of candidate PIM inhibitors **(A)** Four heterogeneous types of evidence were integrated and calculated as the likelihood ratios.   **(B)** ROC curves for evaluating the performances. NB-Loc and NB-Ran denote the Naïve Bayesian model that integrate all the evidence sources and are based on the negative set Loc-NRS and Ran-NRS, respectively. The prediction model is based on the negative reference set Loc-NRS except the NB-Ran model that uses Ran-NRS as the negative reference set. TP/FP ratios (true positive versus false positive) are calculated at different LR cutoffs. **(C)** The PPI of PIM kinases. **(D)** The MTT assay of candidate compounds.

Four heterogeneous types of evidence were integrated and the likelihood ratios were used as the reliability of individual dataset to infer PPIs by using the Naïve Bayesian theorem (Figure 1A). We then used the ROC curve to evaluate the performances of predictions. A protein pair is predicted to be positive when its likelihood ratio is above a particular cutoff, to be negative otherwise (Figure 1B). To get an appropriate composite LR cutoff, we plot the ratio of true positive to false positive (TP/FP) as the function of the cutoff of likelihood ratio. We further modified the globe human PPI into the PIM kinases PPI (Figure 1C). 36 proteins were predicted to interact with PIM1. Such as BMX, CDNIA, MDM2, STAT3 and PTPA are all apoptotic proteins. 14 proteins interacted with PIM2, like apoptotic protein NEMO and all PIM kinases interacted with H90SB and BAD, which were involved in apoptosis. BAD, affecting the level of heterodimerization of Bcl-X(L), Bcl-2 and Bcl-W with BAX, were reported been suppressed Ser112 phosphorylation by PIM kinases. The results showed the creditability of our network. And the PPI network was built for further research of miRNA-PIM-other gene/protein pathways.

### Candidate PIM inhibitor screening and chemical synthesis of PI003

The candidate drugs were carried out by molecule docking, after docking screening the FDA-approved small molecule compounds, 200 top-scored drugs are selected out. From them we chose 20 drugs for further experimentally screening, which were P1-P20 (Supplementary Table S1). P1-P20 are varies from each other in the structure, that means the structural diversity is considered. The MTT assay for P1-P20 showed that P1, P4, P9 and P20 had remarkable inhibitory effects of HeLa cells. Then, we selected out the best one, P9 (Chlorpromazine) (Drugbank number: DB00477) for its best inhibitory effect and capability for further chemical modifications. (Figure 1D).

**Figure 2:**
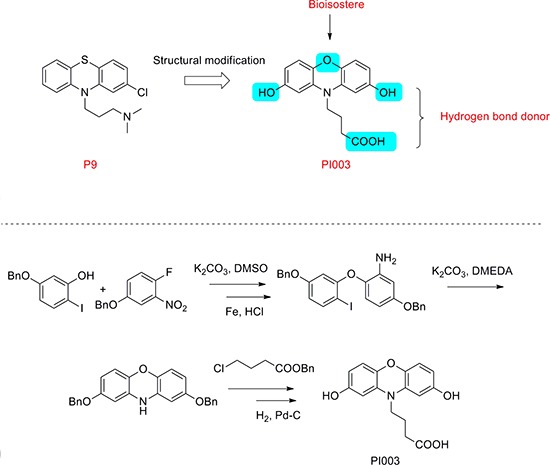
Chemical structure modification strategies of PI003 Compound PI003 was obtained by multi-steps chemical synthesis. The high degree of symmetry in these molecules enabled facile confirmation by NMR techniques.

The synthesis of compound PI003 was shown (Figure 2). 5-(benzyloxy)-2-iodophenol was treated with 4-(benzyloxy)-1-fluoro-2-nitrobenzene and then the reaction was triggered by K2CO3 to obtain the intermediate 4-(benzyloxy)-1-(5-(benzyloxy)-2-iodophenoxy)-2-nitrobenzene in a total yield of 68%. After hydrogenation catalyzed by the Fe/HCl (yield almost 100% without additional purification), the nitro group was reduced into amino. The intermediate 5-(benzyloxy)-2-(5-(benzyloxy)-2-iodophenoxy) aniline was further reacted by K2CO3 and DMEDA to afford the product 2, 8-bis(benzyloxy)-10H-phenoxazine (yield 79%). Then benzyl 4-chlorobutanoate was added to the reaction mixture in present of K2CO3, after final hydrogenation catalyzed by the H2 and Pd-C(yield almost 100%), the compound PI003 was obtained. The high degree of symmetry in these molecules enabled facile confirmation by NMR techniques. The purity of all compounds was above 97.0% determined by HPLC normalization method. Furthermore, the structures of these compounds were further verified by ESI-MS. All the spectra displayed a very prominent peak corresponding to the compounds complexed with protons or sodium cation. PI003: ^1^H NMR (400 MHz, CDCl_3_) δ 6.86 – 6.66 (m, 2 H), 6.63 (d, *J* = 2.4 Hz, 1 H), 4.61 (s, 1 H), 3.93 (t, *J* = 11.2 Hz, 1 H), 2.30 (t, *J* = 11.2 Hz, 1 H), 1.89 (t, *J* = 11.3 Hz, 1 H). ^13^C NMR (100 MHz, CDCl_3_) δ 177.25, 153.29, 140.19, 133.58, 119.19, 113.45, 105.28, 49.05, 30.97, 23.77. m/z = 324.0851(M+Na).

### Molecular docking and MD simulations of PIM kinases /PI003 complex

**Figure 3:**
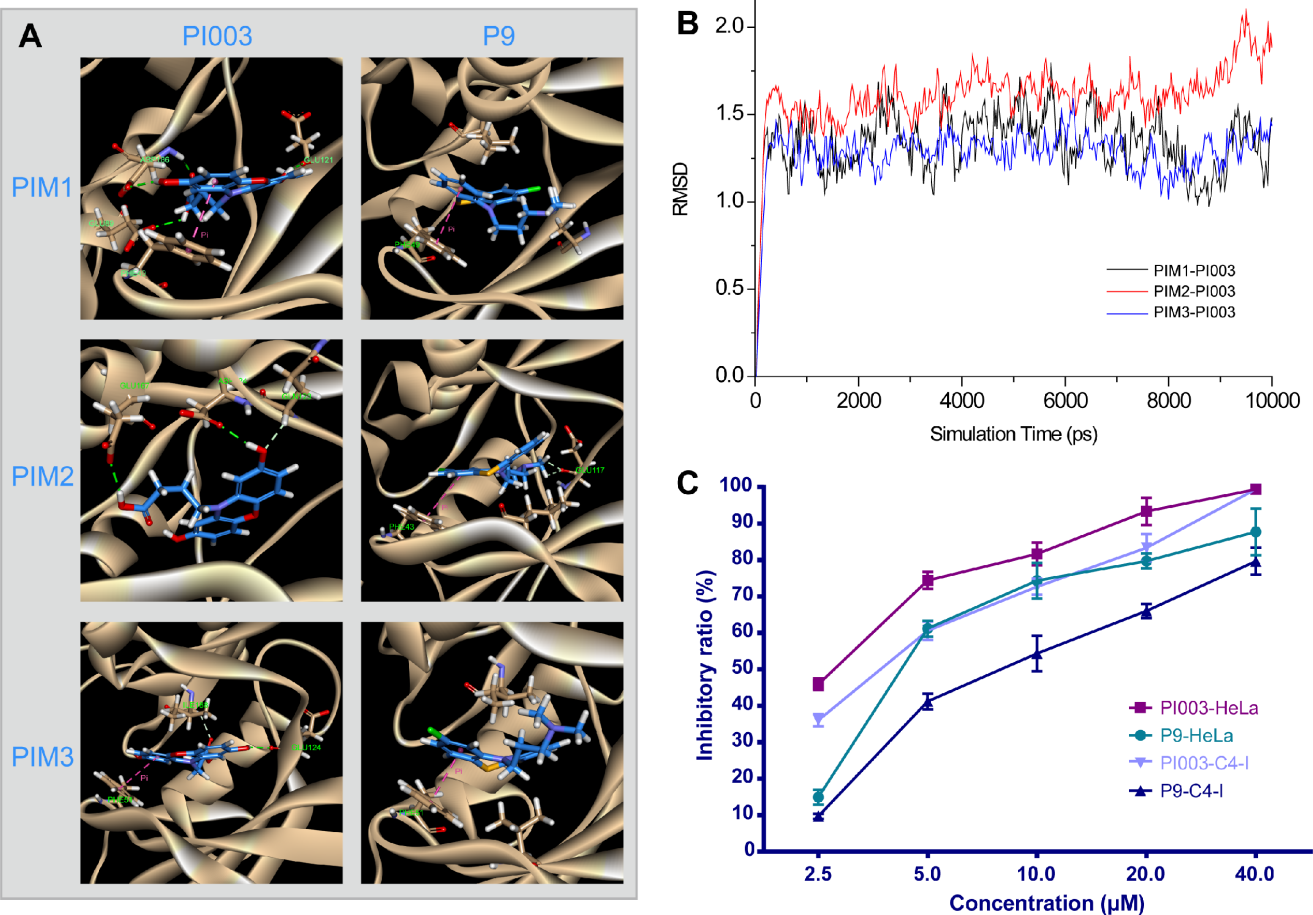
*In silico* and experimental comparisons between PI003 and P9 **(A)** Molecular docking of PI003 and P9. Compared with P9, PI003 has more hydrogen bonds. **(B)** MD simulations of PI003-PIM kinases complexes. The MD simulations were stable after 1 ns for the three complexes. **(C)** The MTT assay of PI003 and P9. The PI003 had a remarkable increase in inhibitory ratio of HeLa and C4-I cells.

PI003 binds to the PIM kinases much better than P9 (Figure 3A). Of note, PIM1, PIM2 and PIM3 are highly homologous at the amino acid level. There is a long and narrow gap in the deep of the active pocket, which is the key side of PIM kinases. PI003 as a “tail” with carboxyl having Hydrogen bonding in the gap. To increase the stability, two hydroxies are added to binding with oxygen atoms outside of the gap. The hydroxies act like the key to the active pocket that brings the good result of MD simulations. As a pan-PIM inhibitor, the docking showed that PI003 target PIM1 better and it can binding to PIM2 and PIM3.

As shown in Figure 3B, the MD simulations were carried out for 10 ns for the three inhibitor-PIM complexes. The RMSD values of PIM1, PIM2 and PIM3 backbone atoms involving the initial minimized structure, which plotted in Figure 3B, were calculated through the phase of the simulation to evaluate the reliable stability of the MD trajectories and the difference of the stabilities in the MD simulations. All of inhibitor-PIM complexes were found to reach equilibrium after 1 ns of the simulation phase. As showed in Figure 3, the RMSD values read 1.2, 1.4 and 1.3 Å for the PIM1, PIM2 and PIM3-inhibitor complexes, respectively, with lower than 0.5 Å deviation. The study demonstrated that the trajectories of the MD simulations were stable after 1 ns for the three complexes, then the binding free energy calculation and free energy decomposition could be carried out on base of the snapshots extracted from 1 to 10 ns.

The binding free energies had been calculated through the MM/GBSA method with the aid of the single trajectory protocol. For analysis of the binding free energy, the 450 snapshots were extracted at a time interval of 20 ps from the 1 to 10 ns of MD trajectories. The calculated binding free energies and components were shown (Table 1). It showed that the binding free energies of PIM1, PIM2 and PIM3-inhibitor complexes were −43.69, −38.81 and −42.12 kcal/mol, respectively. From Table 1, the intermolecular van der Waals and the electrostatics interactions both play an important role in binding, but polar salvation terms act oppositely. Nonpolar solvation terms, corresponding to the burial of SASA upon binding, are moderately favorable. In order to further investigate the influence of the configuration on the hydrogen bonding network, the visible percentage of hydrogen bonds during the MD simulations was calculated and the results was displayed (Table 1 and Table 2). The inhibitory ratio of HeLa and C4-I cells treated with PI003 and P9 were carried out by MTT analysis and the effect of PI003 gained remarkably both in HeLa and C4-I cells (Figure 3C). The IC50 of PI003 were 3.23 μM and 5.38 μM in HeLa and C4-I cells, respectively. And the IC50 of P9 were 6.34 μM and 10.97 μM. The inhibitory ratio of HeLa cells is higher than C4-I cells, thereby HeLa cells were selected for further studies.

**Table 1:** Binding free energies and individual energy terms of PI003 in complex with PIM-1, PIM-2 and PIM-3 (kcal/mol)

Contribution	Pim1-PI003	Pim2-PI003	Pim3-PI003
${\text{ΔE}}_{\mathrm{int}}^{ele}$	−41.60(0.88)	−41.83(0.99)	−34.00(0.54)
${\text{ΔE}}_{\mathrm{int}}^{vdw}$	−34.71(0.37)	−37.57(0.29)	−35.85(0.38)
${\text{ΔG}}_{sol}^{nopol}$	−5.54(0.02)	−5.12(0.02)	−5.60(0.04)
${\text{ΔG}}_{sol}^{ele}$	48.18(0.65)	55.91(0.85)	41.75(0.56)
ΔG_*sol*_^a^	42.64(0.66)	50.79(0.83)	36.15(0.61)
ΔG_*ele*_^b^	6.58(0.06)	14.08(0.12)	7.75(0.10)
−TΔS	−9.8(0.13)	−10.2(0.18)	−8.2(0.09)
ΔG_*bind*_	−43.69(0.32)	−38.81(0.25)	−42.12(0.26)

a The polar/nonpolar ($\Delta G_{\mathrm{sol}}^{\mathrm{ele}}+\Delta G_{\mathrm{sol}}^{\mathrm{nopol}}$) contributions.

b The electrostatic ($\Delta {\text{E}}_{\text{int}}^{\mathrm{ele}}+\Delta G_{\mathrm{sol}}^{\mathrm{ele}}$ does not explicitly consider entropy contributions. The values in parentheses represent the standard error of the mean.

**Table 2:** Hydrogen bonds analysis of the small-molecule inhibitor PI003 into PIM1, PIM2 and PIM3 binding sites based upon MD simulations

Complex	Donor	Acceptor-H	Acceptor	% Occupied	Distance(Ǻ)	Angle(Degree)
PI003-Pim1	57:OE2	DRG:H37	DRG:O22	99.6	2.620	19.44
	154:OD2	DRG:H35	DRG:O18	42.2	2.679	15.11
PI003-Pim2	80:OD1	DRG:H36	DRG:O19	74.3	2.660	19.31
PI003-Pim3	150:OD2	DRG:H36	DRG:O19	97.3	2.682	16.71
	51:OE2	DRG:H37	DRG:O22	95.2	2.626	27.04
	DRG:O21	150:H	150:N	25.8	2.836	27.93

### PI003 induces apoptosis via the death-receptor and mitochondrial pathways in HeLa cells

**Figure 4:**
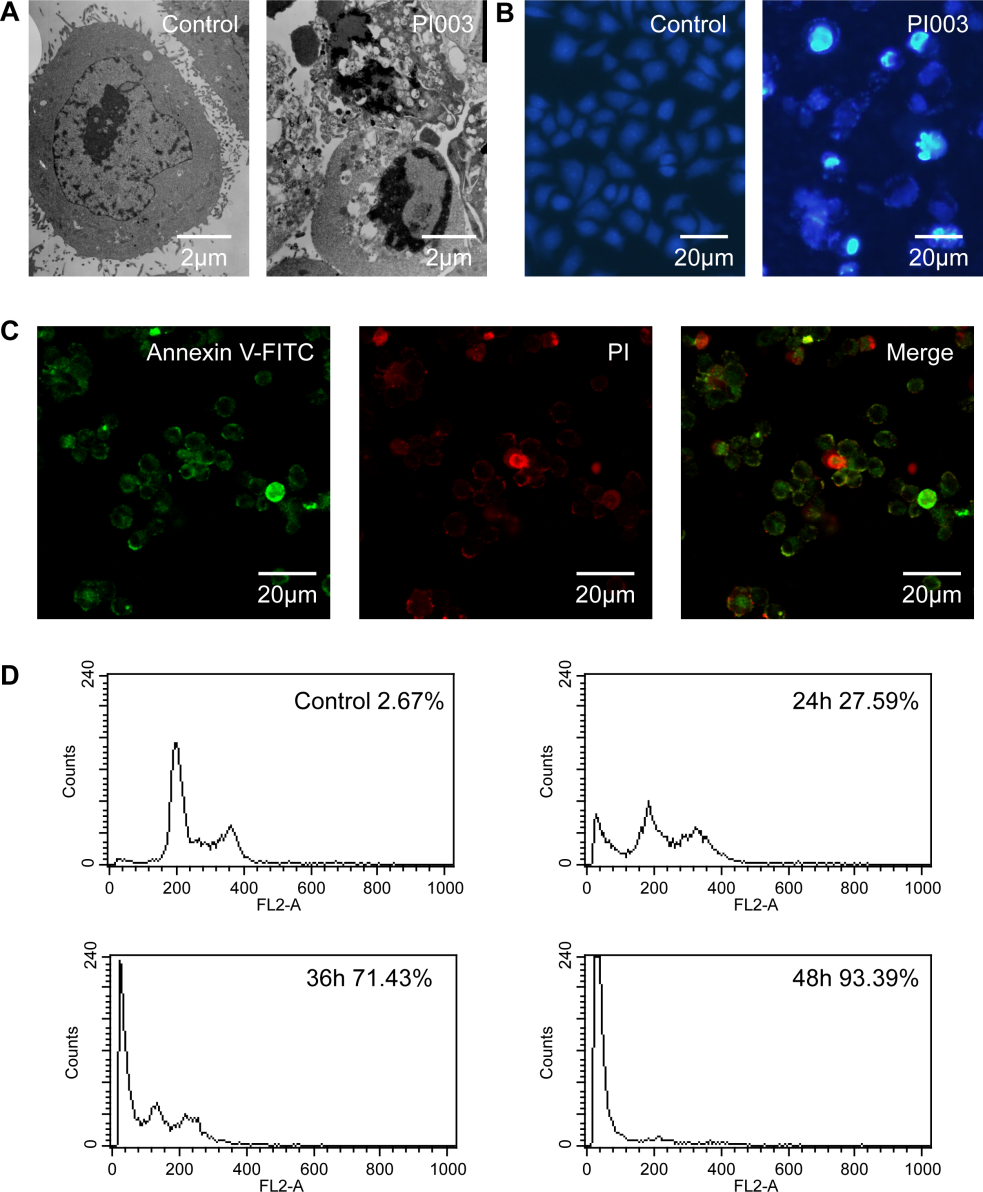
PI003 induces apoptosis in HeLa cells **(A)** The cellular morphology was observed without or with PI003 under the inverted microscopy. **(B)**, **(C)** Apoptosis was determined by the analyses of Hoechst 33342 staining and Annexin staining. **(D)** The population of SubG1 cells was measured by flow cytometry after collection.

Meanwhile, typical characteristics of apoptosis were also observed in PI02-treated HeLa cells. These apoptotic characteristic changes included chromatin condensation and margination at the nuclear periphery (Figure 4A). Hoechst 33342 staining also showed a higher degree of apoptosis in the HeLa cells created with PI003 compared with the negative control (Figure 4B). When PI003 was added, the change of subcellular localization of phosphatidylserine was observed under fluorescence microscope, which is a typical apoptotic feature (Figure 4C). With remarkable reduce of DNA content, the percentage increasing of HeLa cells death also measured by flow cytometry (Figure 4D). Under the microscope, typical apoptotic manifestation are observed.

**Figure 5:**
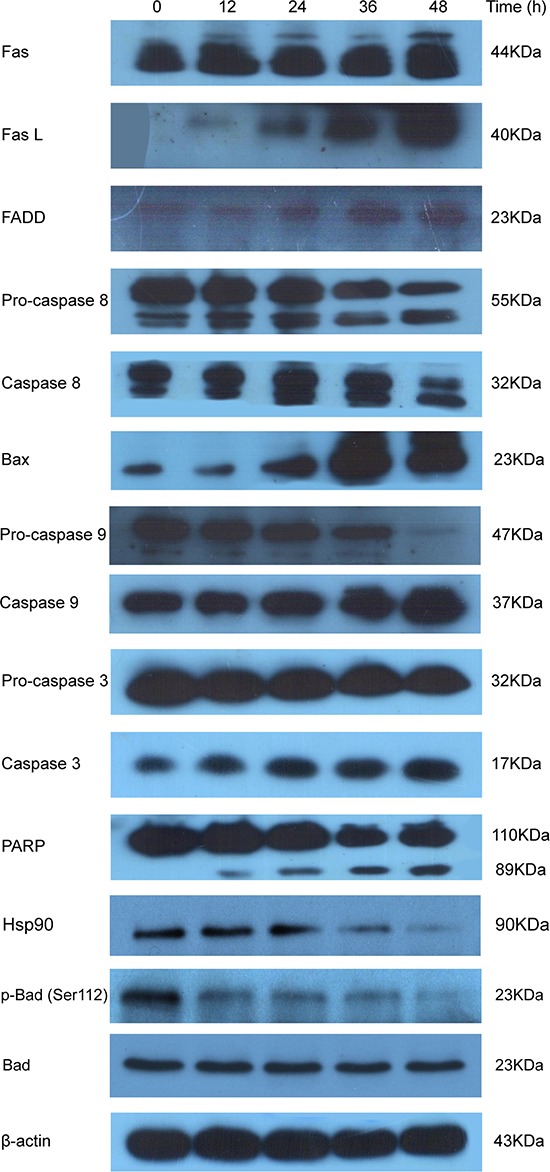
PI003 induces HeLa cell apoptosis via the death-receptor and mitochondrial pathways

Moreover, we examined PI003 induced HeLa cell apoptosis via both death-receptor and mitochondrial pathways by western blot analysis (Figure 5). After treatment with PI003, the levels of Fas, Fas L and FADD were increased. For caspase family, pro-caspase 8 and pro-caspase 3 were decreased, whereas the levels of caspase 8 and caspase 3 were increased. PARP, substrate of caspase 3, was observed a time-dependent cleaving in PI003-treated HeLa cells. Then, we investigated the involvements of Bax, pro-caspase 9 and caspase 9 in PI003-induced apoptosis. These results suggest that PI003 induces HeLa cell apoptosis via the death-receptor and mitochondrial pathways. Interestingly, in the good agreement with above-mentioned prediction (Figure 1C), we found that p-Bad and Hsp90 were both decreased in PI003-treated HeLa cells, indicating that PI003 may be targeting PIM1/2/3-p-Bad/Hsp30 pathway in cancer cells.

### PI003-induced apoptotic mechanism is mainly dependent on PIM-1, but partially dependent on PIM-2 and PIM-3

**Figure 6:**
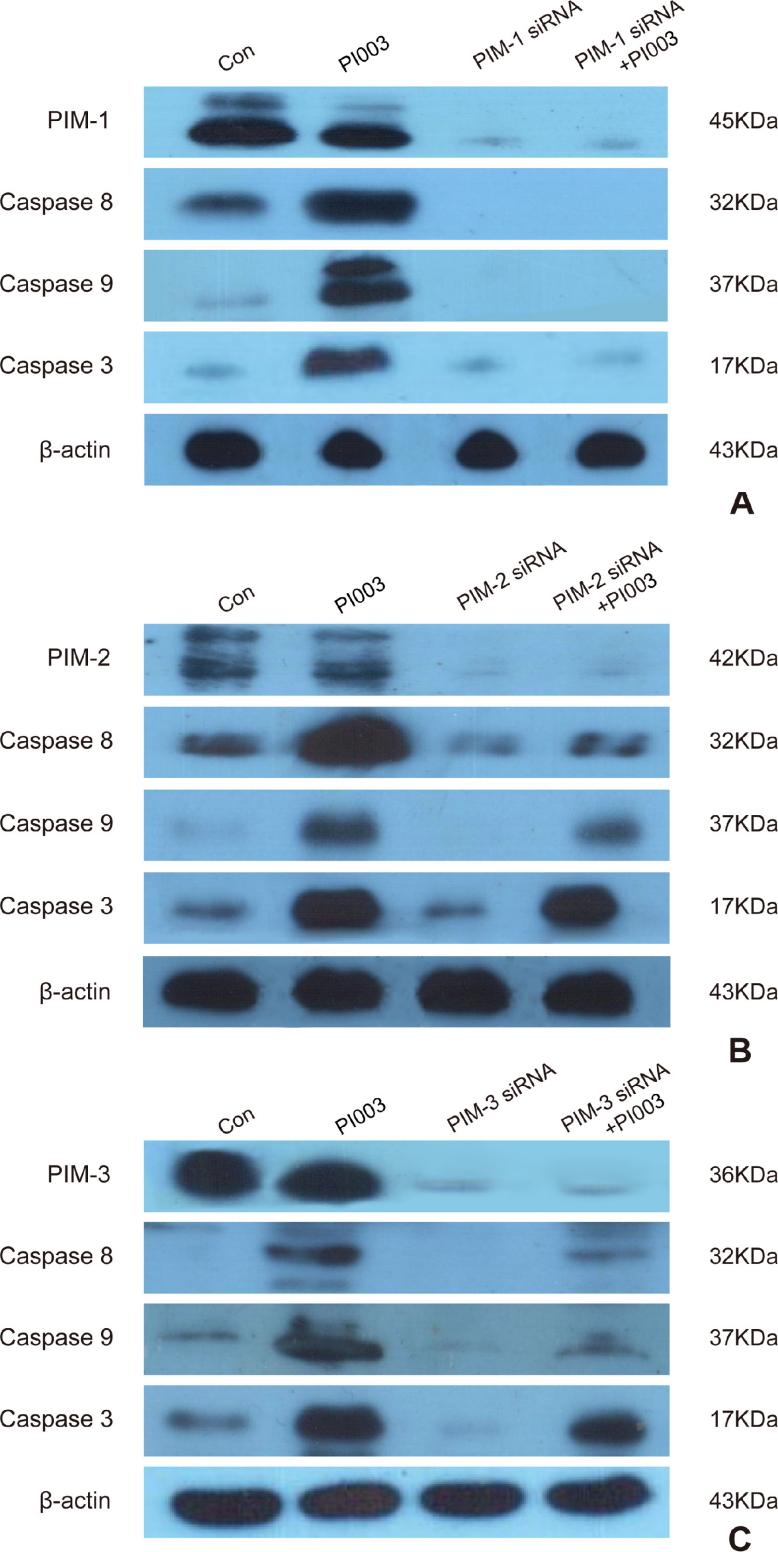
PI003 induces apoptosis by targeting PIM1, PIM2 and PIM3 **(A)** PI003-induced apoptosis is mainly dependent on PIM1; **(B)** PI003-induced apoptosis is partially dependent on PIM2; **(C)** PI003-induced apoptosis is partially dependent on PIM3.

As a pan-PIM inhibitor, PI003 may target PIM1, PIM2 and PIM3. To our best knowledge, PIM1 takes the main effect of PIM kinases in cancer. So the PI003 was designed to target PIM1 mainly (Figure 6A). When treated with PI003, caspase 3, 8 and 9 level showed remarkable increasing and the level of PIM1 decreased. But when PIM-1 siRNA added, PI003 cannot increase the level of caspase 3, 8 and 9. When adding PIM-2 siRNA (Figure 6B) and PIM-3 siRNA (Figure 6C), PI003 partially increased caspase 3, 8 and 9. Thus, these results suggest that PI003-induced apoptotic mechanism is mainly dependent on PIM-1, but partially dependent on PIM-2 and PIM-3.

### MiR-1296/miR-1299 could affect PIM1 and STAT3 in PI003-indcued apoptosis

**Figure 7:**
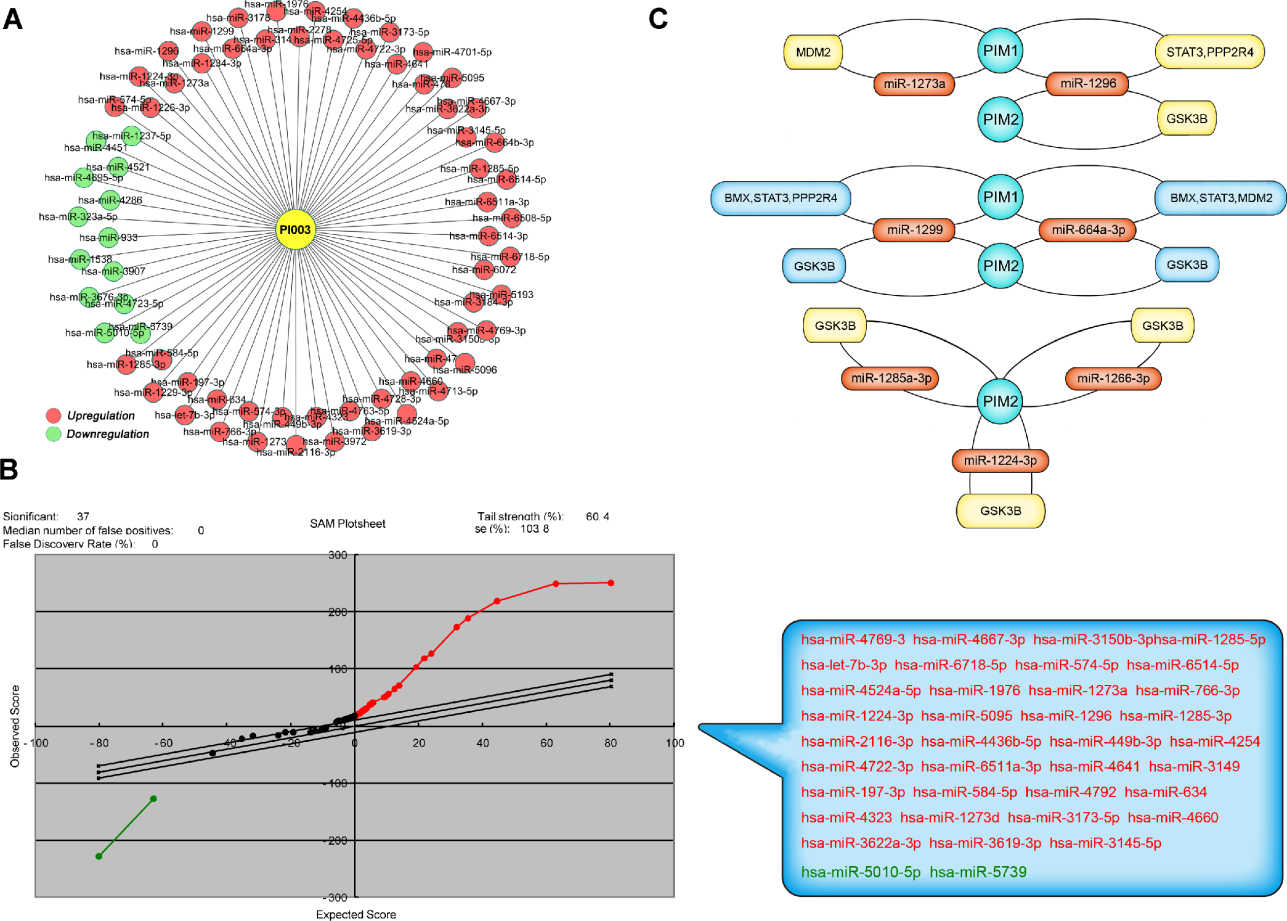
Microarray-based analysis of PI003-induced apoptotic mechanisms involved in microRNAs **(A)** MiRNA microarray analysis of PI003-induced HeLa cell apoptosis. **(B)** The SAM analysis was used to compare the expression data, 37 miRNAs were identified with statistical significance in the treated samples. **(C)** The key-interacted genes in PIM-miRNA-gene/protein apoptotic pathways.

We utilized a miRNA microarray analysis to identify miRNAs that were expressed in HeLa cells when treated with PI003 and 70 miRNAs were identified (Figure 7A). Based on the differentially expressed miRNAs, a SAM analysis was used to compare the expression data of two treated samples and two normal samples (Figure 7B). A total of 35 upregulated and 2 downregulated miRNAs were identified with statistical significance in the treated samples.

**Figure 8:**
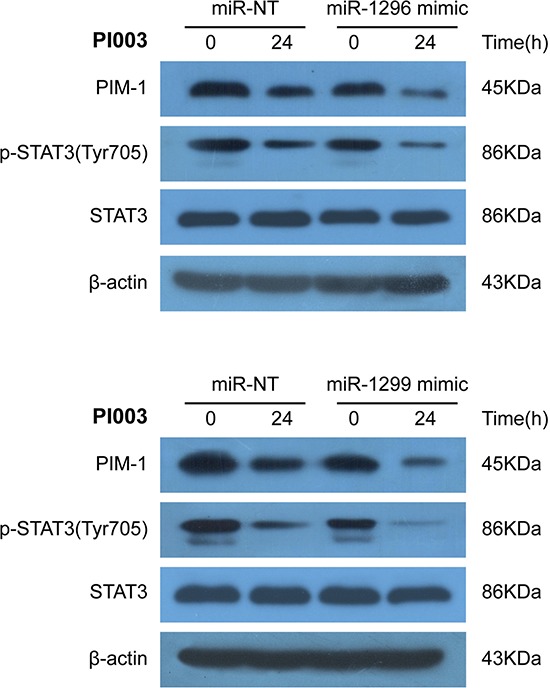
Identification of miR-1296- and miR-1299-regulated mechanisms in PI003-induced apoptosis MiR-1296 mimetic and miR-1299 mimetic could remarkably decrease the expressions of PIM1 and p-STAT3 in PI003-treated HeLa cells.

Seven high conforming predicted miRNAs were selected out, Combined with apoptic proteins in the PPI of PIM kinases, the potential miRNA-PIM-gene pathways were predicted. MiR-1273a, miR-1296, miR-1299 and miR-664a-3p for PIM1 and miR-1273a, miR-1296, miR-1299, miR-664a-3p, miR-1285–3p, miR-1226–3p and miR-1224–3p for PIM2 were selected. And the interacted genes which may play a role in PI003-induced apoptosis and miRNA-PIM-gene/protein pathways were selected (Figure 7C). Such as GSK3B, which has pro-apoptotic effect through phosphorylation of the anti-apoptotic protein MCL1, may control cell apoptosis in response to growth factors deprivation. To further study miRNA involvements, we found that miR-1296 mimetic and miR-1299 mimetic could remarkably decrease the expressions of PIM1, suggesting that they negatively regulates PIM1 and induces apoptosis in HeLa cells (Figure 8). In addition, in PI003-treated HeLa cells, we found that miR-1296 and miR-1299 mimetics could remarkably decrease the expression of p-STAT3, which has been predicted to interact with PIM1 Figure 8. Thus, these results indicate that the two miRNAs can target PIM1-mediated apoptotic pathway in HeLa cells.

### Anti-tumor activity and apoptosis-inducing effect of PI003 *in vivo*

**Figure 9:**
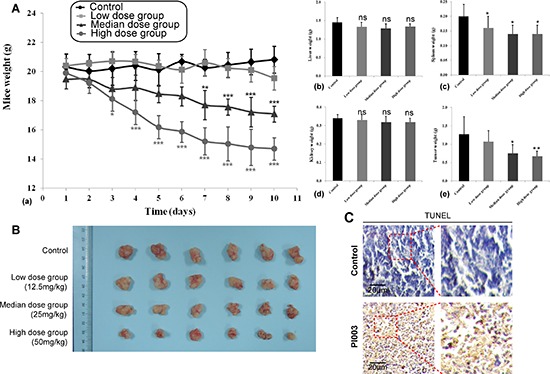
Anti-tumor activities of PI003 in *in vivo* mouse models **(A)** Anti-tumor activities of PI003 and its toxicity. The treatments began on day 1 after grouping (day 0), including vehicle, low dose of PI003 12.5 mg/kg once a day, median dose of PI003 25 mg/kg once a day and high dose of PI003 50 mg/kg once a day for 10 days; **(B)** The inhibitory rate of tumor. Representative tumors from mice after vehicle and PI003 treatment; **(C)** TUNEL immunohistochemistry in representative tumor section of a control mouse and a mouse of the median dose group.

Based on the anti-proliferative efficacy of PI003 on HeLa cells *in vivo*, we proceeded to assess its efficacy on inhibiting tumor growth in an orthotopic xenograft mouse model of cervical cancer. In this experiment, we used three different doses of PI003. Compared with the control group, median and high doses of PI003 can induce the significant body weight loss in nude mice in a dose dependent manner (*P* < 0.001), while the toxicity of low dose PI003 was not obvious (Figure 9A). At the end of the experiment, the tumor weights decreased remarkably in median and high dose groups (median dose group, *P* < 0.05; high dose group, *P* < 0.01). For more toxicity study, spleen weights were affected by different doses of PI003 (*P* < 0.05), the decrease of liver and kidney weights were not obvious. We obtained identical results by directly measuring the tumor volumes. In the three PI003 groups, the tumor volumes were much smaller than the control group in all three doses groups (Figure 9B). In according to the balance between anti-tumor efficacy and toxicity, the median dose was used as the optimum dose for treatment of tumor growth. In addition, PI003 administration resulted in a statistically significant increase in number of apoptotic bodies in the tumor as visualized by TUNEL assay, suggesting that PI003-induced tumor cell proliferation inhibition by apoptosis (Figure 9C).

## DISCUSSION

PIM kinase inhibitors were frequently reported as a new class of cancer therapeutics recently. PIM kinase are regulated primarily by transcription and stability through pathways that are controlled by JAK/STAT, transcription factors. They are interesting targets for new drug development for the overexpressing in many cancers and involving in cancer-specific pathways, such as cell survival, cell cycle progression and cell migration. It has being reported that increased expression of PIM contributed to increasing the susceptibility of leukemia, lymphoma, hepatocarcinoma and prostatic carcinoma. PIM kinase inhibitors are emerging as a new class of cancer therapeutics, and the prevalence of increased PIM kinases expression across different cancer types suggests that PIM inhibitors may be a treatment modality for a variety of cancer [14]. There no research of PIM inhibitors for cervical cancer reported to date. It is a challenge to discover inhibitors of PIM kinases in cervical cancer therapeutics. When PIM1 was inhibited, PIM2 expression increased across different cancer types generally, and Pim-3 was reported to be aberrantly expressed in colon cancer [15]. Taken together, designing pan-PIM inhibitor was necessary for Pim-related cancer therapeutics.

In our study, we developed the Naïve Bayesian model, which was well-suited to integrate these high-throughput data such as smallest shared biological processes (SSBP), gene co-expression profiles, domain-domain interaction (DDI) and cross-species interolog mapping for predicting protein functional connections; thereby, constructing the PIM kinase network. It is well-known that Naïve Bayesian model is used for constructing the global PPI network, suggesting that this mathematics model is advantageous for elucidating the global PPI network in model organisms. Compared to previous studies for the discovery of pan-PIM compounds, the mechanism of PIM inhibitors induced apoptosis was further identified. We identified more novel interacted proteins with PIM kinases (PIM1, PIM2 and PIM3) and further shed light on more molecular mechanisms of PIM kinases in cancer. The other notable result is the binding mode of PI003. Generally, the inhibitors bind to the active site of AMP (Supplementary Figure S1), while in PIM1-PI003 complex, PI003 had Pi interaction with PHE49 and hydrogen bond interaction with GLU89. This binding mode can be used to aid in the design of specific inhibitors of PIM inhibitors.

In this study, FDA-approved small-molecule compounds were screened for candidate drugs and PI003 was modified from the best one, P9, and more hydrogen bonds were formatted to increase the stability. Compared with other pan-PIM inhibitors, such as 7-(4H-1,2,4-Triazol-3-yl)benzo[c] [2, 6] naphthyridines and pyrazolo[1,5-a]pyrimidine-based Pim inhibitors PI003, modified from a FDA-approved drug, had more druggability [16, 17]. A series of experimental data have demonstrated that PI003-induced apoptosis was the death-receptor and mitochondrial pathways in HeLa cells. ETP-45299, which was previously described as a PIM1 inhibitor in various human tumor cells was not tested in HeLa cells. While the PI003 was firstly desired pan-PIM inhibitor in HeLa cells [13]. The evidence was clearer than single PIM kinase inhibitory activity analysis of other PIM inhibitors, such as benzylidene-1,3-thiazolidine-2,4-diones [18]. As the results of western blot analysis, apoptosis via both death-receptor and mitochondrial pathways was identified. Compared with examined the phosphorylation of BAD, like the CX-6258 [19] and LGB321 [20], we clearly explained that the apoptotic mechanisms of PI003. Of note, Pan-PIM inhibitors are valuable for cancer therapeutics. PIM1, PIM2 and PIM3 had different sensitivity of PI003, this design combined the pan-PIM inhibition and low cytotoxicity. Compared with SGI-1776 [21] and AZD1208 [22], in PI003-treated HeLa cells, some new miRNA-regulated mechanisms were also reported. The high differentially expressed miRNAs from microarray with predicted effect of target PIM kinases may play a key role in regulating PIM signaling pathway. The predicted miRNA-PIM-gene pathways, contended the validated interacted proteins of PIM kinases. This results were carried out for the first time and may be experimentally verified in miRNA level. For instance, PIM2 target miRNA, miR-1226–3p, which was verified by NGS (http://mirtarbase.mbc.nctu.edu.tw/), was in one of our predicted miRNA-PIM-gene pathways. For instance, we predicted miR-1296/miR-1299-PIM1-STAT3 pathway, and thus validated the two miRNA mimetic to inhibit the expressions of PIM1 and p-STAT3 in PI003-induced apoptosis of the HeLa cells. This work may shed light on the further research on the combinations of pan-PIM kinases and miRNA drugs in future cancer therapy. Moreover, in our study, we also found that PI003 bear the good anti-tumor activities, and also induced apoptosis *in vivo*. It may provide a board perspective for utilizing PI003 as a potential anti-tumor drug targeting PIMs in the near future.

In conclusion, these findings demonstrate that PI003, as a novel synthesized pan-PIM inhibitor, induces the death-receptor and mitochondrial apoptosis involved in some microRNA regulation, and also possessed remarkable anti-tumor activity and apoptosis-inducing effect *in vivo*. Thus, these findings would provide us a new clue on discovering more potential small-molecule pan-PIM inhibitors in future cervical cancer therapy.

## MATERIALS AND METHODS

### Network construction

To build the protein-protein interaction (PPI) network of PIM kinases, we collected a diverse of datasets from online databases. The globe network data were from Human Protein Reference Database (HPRD) [23], Biomolecular Interaction Network Database (BIND) [24], the Database of Interacting Proteins [25], Munich Information center for Protein Sequences [26], IntAct [27] and PrePPI [28]. Then, we applied a Naïve Bayesian model to integrate diverse data and thus making the final interaction predictions in a systematic way [29]. Following the Naïve Bayesian theorem, we compute the posterior odds given n evidence as follows:

$$O_{\text{posterior}}=\frac{P(\text{positive}|E_{1},...,E_{n})}{P(\text{negative}|E_{1},...,E_{n})}$$

Where *positive* means that two proteins are functional related while *negative* means not.

We define ${\text{LR}}_{\left(E_{1},...,E_{n}\right)}=\frac{P(E_{1},...,E_{n}|\text{positive})}{P(E_{1},...,E_{n}|\text{negative})}$ then *O*_posterior_ = *O*_prior_ × LR.

As Naive Bayesian model supposes that each evidence is conditional independent, we can simplify LR as

$${\text{LR}}_{\left(E_{1},...,E_{n}\right)}=\prod_{i=1}^{n}LR(Ei)$$

A likelihood ratio (LR) corresponding to a specific biological evidence (Ei) was used to measure the predictive power or confidence degree, and calculated as the ratio of the true positive rate (TPR) to the false-positive rate (FPR), where TPR = |Ei⊂PRS|/|PRS| and FPR = |Ei⊂NRS|/|NRS|. In theory, LR (Ei) > 1 indicates that the biological evidence Ei is capable of identifying the true positives from a test. The LR was calculated from four evidences: (A) Interology mapping of 6 model organisms. (B) Gene co-expression data calculated from high quality large-scale microarray datasets. (C) Domain-domain interaction data from PrePPI. (D) Smallest shared biological processes that quantify protein biological process similarity. Based on these evidences, best predicted proteins are chosen by preset scores. And a ROC curve allows us to explore the relationship between the sensitivity and specificity of a binary classifier system for a variety of different cut points [30].

### Molecular docking

FDA-approved small molecule compounds from the latest version of DrugBank (http://www.drugbank.ca/) were downloaded to construct the screening library for PIM kinases. In addition, we used Accelrys Discovery Studio version 3.5 (Accelrys Inc., USA) with CHARMm force-field parameters to dock pre-generated conformations of drugs into PIM1, PIM2 and PIM3 for virtually screening potential candidate inhibitors.  We performed flexible-ligand docking to a rigid receptor with grid-based scoring, in which drugs were allowed to be flexible and structurally rearranged in response to PIM1, PIM2 and PIM3. The P1-P20 were selected based on their scores and structures.

### Cell culture and the MTT assay

The HeLa cells and C4-I cells were cultured in Dulbecco's modified Eagle's medium (DMEM) with 10% (v/v) heat-inactivated fetal bovine serum and incubated in a humidified incubator with 5% CO_2_. HeLa cells and C4-I cells were transferred to 96-well plates at a density of approximately 5.0 × 10^4^ cells/mL. Cytotoxicity induced by the test compounds were measured using the MTT assay as follows: The HeLa cells and C4-I cells were cultured in DMEM in the presence of 30 μM test compound for 24 h and then10ul of MTT (5 mg/ml) was added to cells in each well. After 4 h of culture, the medium was removed, and the blue formazan crystals that had formed were dissolved in dimethyl sulfoxide. The absorbency of formazan generated from MTT was measured at 570 nm (Bio-Rad Model 680, Bio-Rad, Hercules, CA, USA) [31, 32]. The inhibitory ratio (%) = (OD_490, control_ − OD_490,sample_)/(OD_490,control_ − OD_490,blank_)*100.

### Chemical synthesis

All reactions requiring anhydrous conditions were performed under an Ar or N2 atmosphere. Chemicals and solvents were either A.R. grade or purified by standard techniques. Thin layer chromatography (TLC): silica gel plates GF254; compounds were visualized by irradiation with UV light and/or by treatment with a solution of phosphomolybdic acid (20% wt. in ethanol) followed by heating. Column chromatography was performed by using silica gel with eluent given in parentheses. 1H NMR analysis was performed using CDCl3 or DMSO-d6 as a solvent at room temperature. The chemical shifts are expressed in relative to TMS (=0 ppm) and the coupling constants J in Hz. The purity of compound screened in biological assays was determined to be ≧ 97% by HPLC analysis with a photodiode array detector, An atlantis C18 (150mm × 4.6mm, i.d. 5 μm) (Waters, Milford, Mass, USA) was used with a gradient elution of methanol and HPLC-grade water as mobile phase at a flow rate of 1 ml/min [33, 34].

### Molecular dynamics (MD) simulations

MD simulations were performed with GROMACS (version 4.5.5) software package [35] to monitor the binding states between PIM kinases and PI003. The topology parameters of ligands were built by the Dundee PRODRG server [36]. The topology of PIM kinases was edited by Amber force field 99SB and the small molecule was edited by Amber general force field. The complexes were immersed in a cubic box of simple point charge (SPC) water molecules. Eight and eleven sodium counter-ions were added by replacing water molecules to ensure the overall charge neutrality of the receptors simulated system, respectively. In this MD process, 1 ns simulations with a time step of 10 ns were performed, and the resulting trajectory files were viewed and analyzed using VMD software [37].

### Apoptosis detection

The ultrastructure of cell apoptosis was observed under an electron microscope (Hitachi 7000, Japan). Hoechst 33342 staining and Annexin V-FITC staining were performed to detect apoptosis. For Hoechst 33342 staining, A549 cells were washed with PBS and stained with Hoechst 33342 (1 μg/ml in PBS) at room temperature for 20 min, the fluorescence was observed by a fluorescence inverted microscopy. For Annexin V-FITC staining, the treated cells were collected, washed and then stained with Annexin V-FITC or PI at room temperature for 15 min. The percentage of apoptotic cells were analyzed by flow cytometry (Becton Dickinson, Franklin Lakes, NJ) [38–40].

### Western blot analysis

Cellular proteins were extracted using RIPA buffer (SolarBio, 50 mM Tris/HCl, pH 7.4, 150 mM NaCl 1% (v/v) NP-40, 0.1% (w/v) SDS) containing 1% (v/v) PMSF (SolarBio), 0.3% (v/v) protease inhibitor (Sigma) and 0.1% (v/v) phosphorylated proteinase inhibitor (Sigma). Lysates were centrifuged at 12,000 rpm at 4°C for 15 min and the supernatant was collected for total protein. A BCA protein assay kit (Pierce) was used to determine the protein concentration. Equal amounts of protein (15 μg) was separated on an SDS-PAGE gel (10% (v/v) polyacrylamide) and transferred onto a PVDF membrane. Nonspecific binding was blocked using 8% (w/v) milk in TBS-T for 2 hr at room temperature. The membranes were then incubated with primary antibodies overnight at 4°C. After several washes with TBS-T, the membranes were incubated in HRP-conjugated goat anti-rabbit and anti-mouse IgG or HRP-conjugated mouse anti-goat IgG (Abmart, all at a 1:5000 dilution) for 2 hr at room temperature and then washed. The target proteins were visualized using enhanced chemiluminescence (Millipore) according to the manufacturer's recommendations, and quantified using density analysis normalized against GAPDH to the manufacturer's recommendations, and expressed as the fold-change compared to control.

### SiRNA and miRNA transfection

Small interfering RNAs (siRNAs) against human PIM-1, PIM-2 and PIM-3, and control siRNA were purchased from Invitrogen (Carlsbad, CA). MiR-1296 mimetic and miR-1299 mimetic were purchased from Sigma. The HeLa cells were transfected with siRNAs at 33 nM final concentration using Lipofectamine 2000 (Invitrogen) according to the manufacturer's instructions. For the miRNA transfection, HeLa cells were transfected with miR-1296 mimetic and miR-1299 mimetic at 100 nM final concentration using Lipofectamine RNAiMAX reagent (Invitrogen) according to the manufacturer's instructions. The transfected cells were used for subsequent experiments 24 h later.

### MicroRNA microarray and SAM analysis

Human OneArray^®^ microarrays were pre-heated at 60°C for 10 min in hybridization oven. Microarray slides were placed inside a falcon tube containing 100% ethanol, incubated for approximately 15 sec, shaken for 20 sec, and thoroughly rinsed with deionized water to remove any residual ethanol. Next, the microarray slides were fully submerged in an abundant amount of pre-hybridization solution (5X SSPE, 0.1% SDS, and 1% BSA) for 1 hr at 42°C. After 1 hr, slides were transferred to room-temperature distilled water and washed gently for 2 min. Slides were spun dry for 2 min and stored in a dry and dark place until hybridization.

10 μg of cRNA was fragmented by using RNA Fragmentation Reagent kit (AM#8740, Ambion Inc., Austin, Texas, USA), and then denatured in a PCR machine at 95°C for 5 minutes and held at 60°C. Fragmented cRNA was hybridized on the rice OneArray^®^ (Phalanx Biotech Group, Taiwan) at 50°C for 14–16 hrs. After hybridization, the microarrays were washed sequentially in 2X SSC containing 0.2% SDS solution for 5 min at 42°C, 2X SSC for 5 min at 42°C, and 2X SSC for 5 min at room temperature. Finally, the microarrays were spun dry with a centrifuge for at least one minute and stored dry in the dark until ready for scanning [41].

The significant analysis of microarray (SAM) method was used to perform the unsupervised calculation. The statistical technique is based on a *t*-test for finding significant miRNAs in a set of microarray experiments and was proposed [42]. A hierarchical clustering of the differentially expressed miRNAs was performed with Cluster 3.0 (http://bonsai.hgc.jp/~mdehoon/software/cluster/software) version using the average linkage algorithm. The top scoring pair (TSP) algorithm was used to perform the supervised calculation [43]. The basic principle of the k-TSP is to identify miRNA pairs that are oppositely expressed (one upregulated and one downregulated) in two classes. All numerical analyses that are presented were performed using Matlab 7.0 (MathWorks Company, Natick, MA, USA).

Target miRNAs that bound to PIM kinases were predicted using miRWalk, a database on predicted and validated microRNA targets, so did the predicted targets of miRNAs [44]. Data from 9 databases (DIANA-mT [45], miRanda [46], PICTAR5 [47], miRDB [48], PITA [49], miRWalk [50], RNA22 [51], RNAhybrid [52] and TargetScan [53]) was used for identifying the high conforming predicted miRNAs. The potential miRNA-PIM-gene/protein pathways were predicted by the combining of the predicted targets of conforming miRNAs and the apoptotic proteins in PPI network of PIM kinases.

### Mouse experiments and tumor xenograft model

The Institutional Animal Care and Treatment Committee of No.150 Central Hospital of PLA approved all studies herein. 24 healthy female nude mice (BALB/c, 6–8 weeks of age, non-fertile and 18–20 g each) were injected subcutaneously with HeLa cells (1 × 10^7^cells/mouse). When the tumors reached 100 mm^3^ in volume (calculated as V = L × W^2^/2). The mice were divided into four groups. Three groups were treated with different dose groups of PI003 once a day by intraperitoneal injection for 10 days (low dose group, 12.5 mg/kg; median dose group, 25 mg/kg; high dose group, 50 mg/kg), whereas the control group was treated with vehicle control (5% CMC-Na). Body weight was determined every day until the end of the study. At the end of the treatment, all mice were sacrificed. Tumor tissue, spleen, liver and kidney were harvested, weighed, and photographed.

### TUNEL assay

For TUNEL assay, sections were permeabilized with 0.1% Trition X-100 plus 0.1% sodium citrate and then incubated with 50 ml TUNEL reaction mixture (Roche) at 37°C for 60 min. After rinsing with PBS three times, 50 ml converter-POD was added and the tissue cells were incubated in a humidified chamber for 30 min at 37°C. DAB substrate was then added, followed by counterstaining with hematoxylin. The assay included negative controls (without terminal transferase).

### Statistical analysis

All the presented data and results were confirmed in at least three independent experiments. The data are expressed as means ± SD. Statistical comparisons were made by Student's *t*-test. *p* < 0.05 was considered statistically significant.

## SUPPLEMENTARY FIGURE AND TABLE



## Abbreviations

PIM: serine/threonine-protein kinase
LR: a likelihood ratio
TPR: the true positive rate
FPR: the false-positive rate
FDA: Food and Drug Administration
DMEM: Dulbecco's modified Eagle's medium
MTT: 3-(4,5-dimethylthiazol-2-yl)-2, 5-diphenyltetrazolium bromide
MD: molecular dynamics
PMSF: Phenylmethanesulfonyl fluoride
PVDF: polyvinylidene difluoride
SDS-PAGE: polyacrylamide gelelectrophoresis
SAM: significant analysis of microarray
SD: standard deviation
