# Supplementary material for: Computational prediction and experimental validation of a novel synthesized pan-PIM inhibitor PI003 and its apoptosis-inducing mechanisms in cervical cancer
Source: Oncotarget. 2015 Feb 4;6(10):8019–35. doi: 10.18632/oncotarget.3139 (PMC4480732; doi:10.18632/oncotarget.3139)
Supplement: Supplementary file 1 [file oncotarget-06-8019-s001.pdf]

SUPPLEMENTARY FIGURE AND TABLE

```
PIM1 129 YQVGPLLGSGGFGSVYSGIRVSDNLPVAIKHVEKDRISDWGELPNGTRVPMEVVLLKKVS
PIM2  32 YRLGPLLGKGGFGTVFAGHRLTDRLQVAIKVIPRNRVLGWSPLSDSVTCPLEVALLWKVG
PIM3  40 YQVGAVLGSGGFGTVYAGSRIADGLPVAVKHVVKERVTEWGSL-GGATVPLEVVLLRKVG

PIM1 189 S--GFSGVIRLLDWFERPDSFVLILERPEPVQDLFDFITERGALQEELARSFFWQVLEAV
PIM2  92 AGGGHPGVIRLLDWFETQEGFMLVLERPLPAQDLFDYITEKGPLGEGPSRCFFGQVVAAI
PIM3  99 AAGGARGVIRLLDWFERPDGFLLVLERPEPAQDLFDFITERGALDEPLARRFFAQVLAAV

PIM1 247 RHCHNCGVLHRDIKDENILIDLNRGELKLIDFGSGALLKDTVYTDFDGTRVYSPPEWIRY
PIM2 152 QHCHSRGVVHRDIKDENILIDLRRGCAKLIDFGSGALLHDEPYTDFDGTRVYSPPEWISR
PIM3 159 RHCHSCGVVHRDIKDENLLVDLRSGELKLIDFGSGALLKDTVYTDFDGTRVYSPPEWIRY

PIM1 307 HRYHGRSAAVWSLGILLYDMVCGDIPFEHDEEIIRGQVFFRQRVSSECQHLIRWCLALRP
PIM2 212 HQYHALPATVWSLGILLYDMVCGDIPFERDQEILEAELHFPAHVSPDCCALIRRCLAPKP
PIM3 219 HRYHGRSATVWSLGVLLYDMVCGDIPFEQDEEILRGRLLFRRRVSPECQQLIRWCLSLRP

PIM1 367 SDRPTFEEIQNHPWM
PIM2 272 SSRPSLEEILLDPWM
PIM3 279 SERPSLDQIAAHPWM
```

A

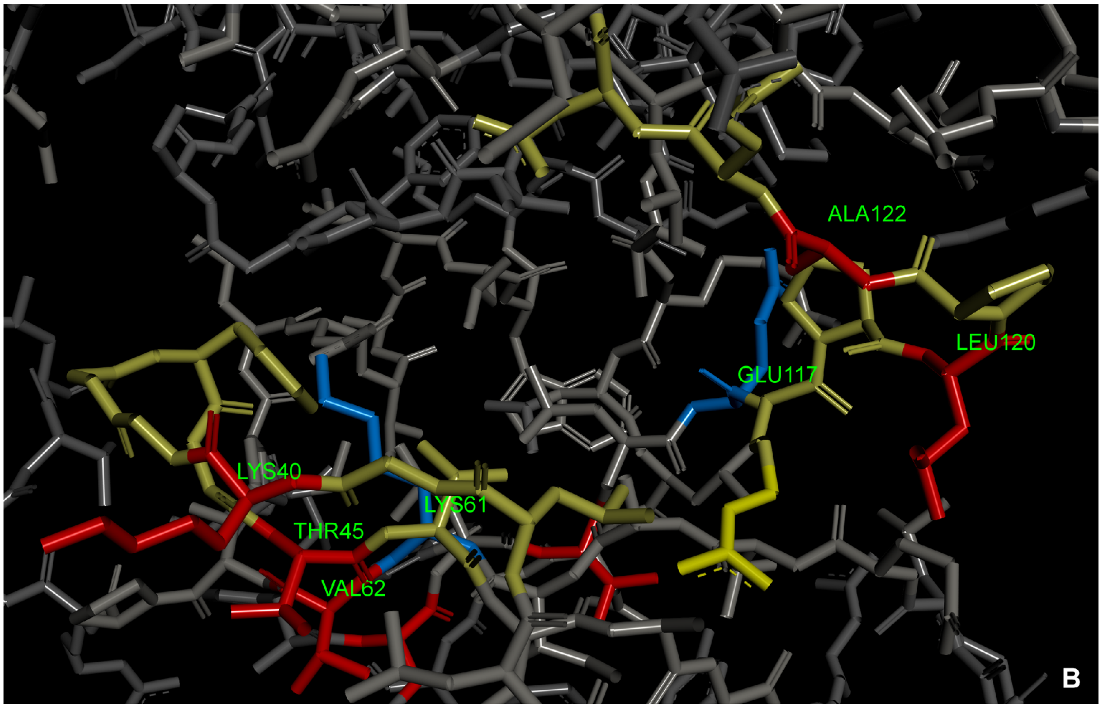

Supplementary Figure S1: Sequence alignment of kinase domains and structure-based differences between PIM1 and PIM2. (A) Sequence alignment of kinase domains of PIM1, PIM2 and PIM3, the differences are underlined with red background or words. The amino acids that form part of the ATP binding are marked with blue boxes; **(B)** The active side (yellow) of PIM2, LYS40, THR45, VAL62, LEU120 and ALA122 (red) are differences in the protein sequence. LYS61 and GLU117 (blue) are amino acids with conformational differences between PIM1 and PIM2, which influence the effect on PIM2 of PIM1 inhibitors with crystallographic structure.

Supplementary Table S1: The MTT assay of candidate small-molecule PIM inhibitors from Drugbank

Number	Name	CAS NO.	Structure	Inhibitory ratio (%)
P1	Carvedilol	72956-09-3	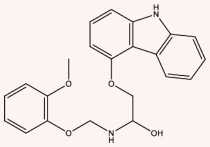	51.77537144
P2	Lansoprazole	103577-45-3	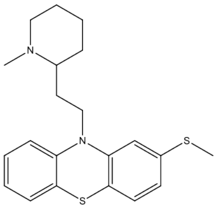	22.66431629
P3	Benserazide hydrochloride	14919-77-8	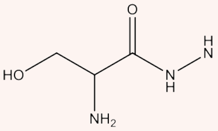	10.12339461
P4	Rosiglitazone hydrochloride	302543-62-0	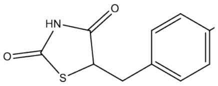	46.81440443
P5	Troglitazone	97322-87-7	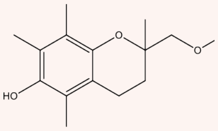	6.925207756
P6	Metformin HCL	1115-70-4	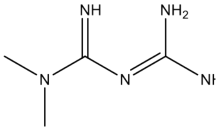	11.20624528
P7	Propranolol Hydrochloride	318-98-9	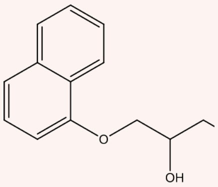	3.878116343
P8	thioridazine hydrochloride	130-61-0	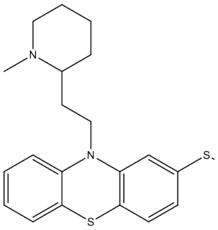	59.12868295
**P9**	**Chlorpromazine**	**50-53-3**	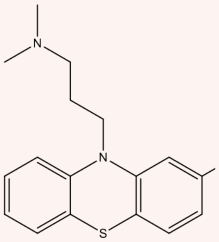	**93.95618232**

(*Continued*)

Number	Name	CAS NO.	Structure	Inhibitory ratio (%)
P10	Hydroxychloroquine sulfate	747-36-4	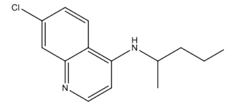	
P11	Primaquine diphosphate	63-45-6	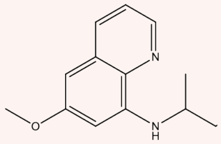	39.56182322
P12	Ethambutol Hydrochloride	1070-11-7	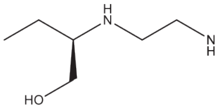	10.75295895
P13	Lincomycin Hydrochloride	859-18-7	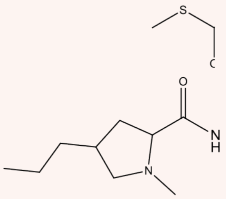	29.84134979
P14	cisapride	81098-60-4	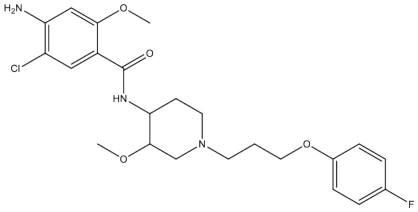	38.10123395
P15	xantinol nicotinate	437-74-1	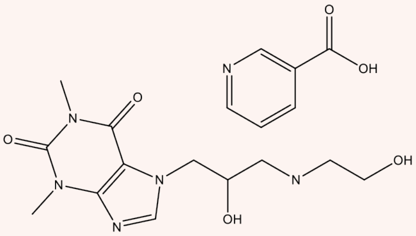	11.45807101
P16	Valaciclovir	124832-26-4	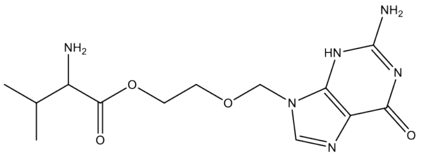	
P17	Fluoxetine hydrochloride	56296-78-7	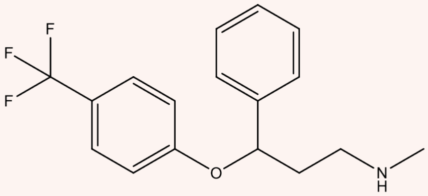	12.36464367
P18	Flavoxate Hydrochloride	3717-88-2	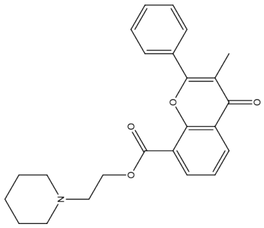	−1.284311257

(*Continued*)

Number	Name	CAS NO.	Structure	Inhibitory ratio (%)
P19	Formoterol Fumarate	43229-80-7	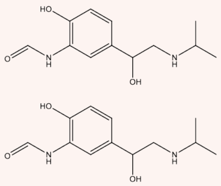	0.138504155
P20	Daunorubicin hydrochloride	23541-50-6	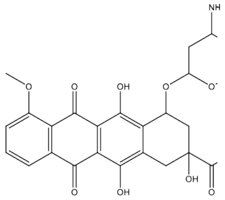	91.26164694
